# Utility of Infrared Thermography for Monitoring of Surface Temperature Changes During Horses’ Work on Water Treadmill with an Artificial River System

**DOI:** 10.3390/ani15152266

**Published:** 2025-08-01

**Authors:** Urszula Sikorska, Małgorzata Maśko, Barbara Rey, Małgorzata Domino

**Affiliations:** 1 Department of Animal Breeding, Institute of Animal Science, Warsaw University of Life Sciences (WULS-SGGW), 02-787 Warsaw, Poland; urszula_sikorska@sggw.edu.pl (U.S.); malgorzata_masko@sggw.edu.pl (M.M.); 2 Scientific Circle of Biotechnologists KNBiotech, Warsaw University of Life Sciences (WULS-SGGW), 02-787 Warsaw, Poland; s227056@sggw.edu.pl; 3 Department of Large Animal Diseases and Clinic, Institute of Veterinary Medicine, Warsaw University of Life Sciences (WULS-SGGW), 02-787 Warsaw, Poland

**Keywords:** equine, hydrotherapy, water counterflow, water treadmill, infrared thermography

## Abstract

Water treadmill (WT) exercise is used for horses’ rehabilitation and training. Given that each training needs to be individualized for each horse, the goal is to assess whether infrared thermography (IRT) can serve as a non-invasive tool for daily monitoring of individual training and rehabilitation progress in horses undergoing WT exercise. Fifteen Polish Warmblood school horses were subjected to five WT sessions: dry treadmill, fetlock-depth water, fetlock-depth water with artificial river (AR), carpal-depth water, and carpal-depth water with AR. IRT images, collected pre- and post-exercise, were analyzed for the mean temperature (Tmean) and maximal temperature (Tmax) across 14 regions of interest (ROIs) representing the body surface overlying specific superficial muscles. While on a dry treadmill, Tmean and Tmax increased post-exercise in all ROIs; wetting of the hair coat limited surface temperature analysis in ROIs annotated on limbs. Tmax over the *m. brachiocephalicus*, *m. trapezius pars cervicalis*, *m. triceps brachii*, and *m. semitendinosus* increased during walking in carpal-depth water, which therefore may be suggested as an indirect indicator of increased activity related to forelimb protraction and flexion–extension of the limb joints. Tmax over the *m. latissimus dorsi* and *m. longissimus* increased during carpal-depth WT exercise with active AR mode, which may be suggested as an indicator of increased workload including vertical displacement of the trunk.

## Simple Summary

Water treadmill exercise is a form of hydrotherapy that is beneficial for both rehabilitation and training and thus used in the equine industry. This study explored the possibility of monitoring horses exercising on water treadmills by using infrared thermography, an indirect modality of measuring underlying muscle work and local blood flow. This study aims to determine whether the temperature of the body surface overlying specific muscles changes during water treadmill exercise depending on training type. Fifteen horses were exercised on dry and water treadmills at varying water depths and under varying workload created by an artificial river. Infrared images were captured before and after each exercise, and the maximal temperature and mean temperature of body surface areas overlying specific muscles were measured. While both temperature measures increased with exercise, their measurement on limbs is hindered by a wet hair coat. An increase in the maximal temperature over specific muscles located on the neck and upper parts of limbs may indirectly indicate workload during walking in carpal-depth water. Similarly, an increase in the maximal temperature over specific trunk muscles may indirectly indicate workload during walking in carpal-depth water with an active artificial river. This method may potentially help trainers and veterinarians track how horses respond to water treadmill exercise.

## 1. Introduction

Water treadmill (WT) exercise is a form of hydrotherapy [1] used in the equine industry for both rehabilitation [2,3,4,5] and training purposes [6,7,8,9,10]. Focusing on rehabilitation, Tranquille et al. [2], through a survey-based study, reported that WT is commonly incorporated into rehabilitation programs for cases such as suspensory and tendon injuries, back, hindlimb, and hoof diseases, post-surgical recovery, and reintroduction to work following arthritis or colic. Potenza et al. [3], using veterinary medical records, found that horses undergoing WT rehabilitation after arthroscopic surgery of the metacarpophalangeal/metatarsophalangeal and carpal joints were more likely to return to racing compared to those receiving conventional rehabilitation. However, only King et al. [4,5], in two studies, have provided objective evidence of rehabilitation progress following experimentally induced carpal joint osteoarthritis—assessing postural sway [4] and limb kinematics [5].

Focusing on training purposes, Greco-Otto et al. [6] demonstrated that incorporating WT protocols into training programs over a 4.5-week period improved peak oxygen consumption in horses, resulting in a beneficial conditioning effect. However, this effect remains within the aerobic range, as WT exercise is considered low-intensity aerobic activity [6,11,12,13,14]. Fair et al. [7], after examining horses trained on a WT for 4 weeks, observed growth of the thoracic back profile musculature (*m. longissimus*). Similarly, de Meeûs d’Argenteuil et al. [8] found that 8 weeks of WT training led to increased muscle diameter in selected regions, including the neck and back (*m. brachiocephalicus*, *m. trapezius pars cervicalis*, and *m. trapezius pars thoracica*), forelimb (*m. biceps brachii* and *m. triceps brachii*), and hindlimb (*m. quadriceps femoris*, *m. biceps femoris*, *m. gluteus medius*, *m. semitendinosus*, and *m. semimembranosus*). Murray et al. [9], examining horses after 20 weeks of WT training, reported muscle development in the thoracic region (*m. trapezius pars thoracica*), lumbosacral region, and hindlimb region (*mm. gluteii*, hamstring muscles, and hindlimb adductor/abductor muscles). In horses, the largest muscle in the lumbosacral region is the *m. longissimus*, while the primary hamstring muscles are the *m. biceps femoris*, *m. semitendinosus*, and *m. semimembranosus* [15]. Nankervis et al. [10], by examining horses trained on a WT for 40 weeks, demonstrated that regular WT exercise leads to changes in gait technique during WT walking. These changes may be beneficial for sport horse training programs aimed at achieving specific training goals. In this and previous studies, Nankervis’s research group has shown that WT exercise induces significant kinematic adaptations compared to walking on a dry treadmill [10,16,17,18]. At relatively low water depths—up to the level of the carpal joint—this and other research has reported increased flexion of the distal limb joints [10,19,20,21], increased forelimb protraction and decreased forelimb retraction [10,20], increased hindlimb protraction and decreased hindlimb retraction [10,17], enhanced flexion–extension of the thoracolumbar spine [10,18,22] and pelvis [10,23], as well as greater vertical displacement of the poll, withers, sacrum, and pelvis [5,10,18,21,22,23].

Nankervis et al. [10] emphasized that the response to WT exercise depends on the type and frequency of the exercise sessions, as well as the horse’s prior experience with WT training. Regarding the type of WT exercise, recent studies have shown that increased extension and protraction of the forelimbs and hindlimbs against the resistance posed by water lead to increased muscle work. To date, most research has identified water depth [6,10,13,16,20,21,24,25,26] and belt speed [6,13,16,25,26] as the primary factors contributing to drag that resists forward limb movement. However, only one study has also investigated the additional resistance generated by water counterflow created by an artificial river (AR) [14]. Our previous work demonstrated that, at relatively low water depths, the intensity of WT exercise with an AR increased progressively as the water level rose from the fetlock to the carpal joints, while still remaining within the low-to-moderate aerobic range [14]. Nankervis et al. [10] also stressed that every component of training—including WT exercise—should be tailored to address the horse’s individual training goals. Therefore, developing more informed training protocols that integrate various combinations of water depth, belt speed, session duration and frequency [10], and potentially AR use [14] should be a key objective in future research.

However, since horses may vary significantly in their individual responses to WT exercise [18,21,22], it is essential to implement daily monitoring of each horse’s training progress. To date, horse workload during WT exercise has been assessed using heart rate (HR) [6,11,12,13,14,26,27,28,29], blood parameters [6,11,13,14,28,29,30], and spirometry [6,13], with HR monitoring being the only method readily applicable in daily practice. Meanwhile, horses’ response to exercise during WT sessions has been primarily evaluated through kinematic analysis [2,4,5,10,16,17,18,19,20,21,22,23,24,25,26] and, in select studies, through surface electromyography (sEMG) [5,31], muscle morphometrics [7,8], as well as infrared thermography (IRT) [32]. Despite the valuable insights provided by kinematic analysis, its practical application in daily use remains limited. As a result, there is growing interest in the use of IRT to monitor horses during WT exercise.

IRT can detect changes in body surface temperature, directly reflecting metabolism and local blood flow of underlying tissue [33,34]. On one hand, heat is a by-product of muscle contraction [35]; therefore, positive correlations have been reported between surface temperature and muscle work, including exercise duration [36], exercise intensity [37,38], and workload in horses [39,40]. On the other hand, heat is also generated by increased blood flow [33,34], for example, when blood flow rises in muscles to meet the metabolic demands of contracting fibers [41]. This increased blood flow is a physiological effect of aerobic exercise on circulation and is particularly relevant for potential daily monitoring of individual training and rehabilitation programs involving WT use. WT exercise is generally classified as aerobic workload [6,11,12,13,14]. IRT was therefore expected to serve as an indirect modality of measuring muscle work and local blood flow during WT exercises [32]. To date, the only relevant study that combined IRT and conventional WT measured the maximum temperature (Tmax) of the body surface overlying only one muscle (*m. semitendinosus*) from the posterior–anterior view. The authors showed the significant difference in measurements taken on dry and water treadmills and indicated the need for further work on IRT applications during WT exercises [32]. To fill this gap, this study aims to determine whether the two measures of surface temperature (Tmax and mean temperature (Tmean)) overlying 17 superficial muscles from the lateral view change during conventional WT exercise and WT exercise under varying workload created by AR. We hypothesize that the surface temperature relatively increases during WT exercise and may thus serve as an indirect indicator of physiological response to exercise. The goal is to assess whether IRT can be further investigated as a non-invasive tool for daily monitoring of individual training and rehabilitation progress in horses undergoing WT exercise.

## 2. Materials and Methods

### 2.1. Animals

Horses were recruited from the head at the Didactic Stable of the Horse Breeding Division at the Warsaw University of Life Sciences (WULS). The inclusion criteria were: no prior experience with water treadmill exercise, no history of orthopedic disease, no history of poor performance, no clinical signs of disease, and no lameness. The histories were obtained from the training and veterinary records of the Didactic Stable. For horses without a history of water treadmill exercise, orthopedic disease, or poor performance, a basic physical examination was conducted. During this examination, the rectal temperature, heart rate, respiratory rate, mucous membranes, capillary refill time, and lymph nodes were assessed. Horses that showed no clinical signs of disease—defined as all physical parameters within physiological ranges [42]—underwent an orthopedic examination according to the American Association of Equine Practitioners (AAEP) lameness scale [43].

Fifteen school horses (n = 15) with a lameness score of 0/5 [43] were included in the study (8 geldings, 7 mares; age range: 6–18 years; median age: 12 years; wither height range: 152–168 cm; median height: 166 cm). All horses were of the Polish Warmblood type, including ten Polish Halfbred horses and five Malopolska breed horses. All horses were housed under the same conditions at the Didactic Stable of the Horse Breeding Division at WULS. They were fed individually. The hay, oats, and concentrate ratios were calculated for each horse, considering the individual nutritional requirements. Horses had constant access to fresh water, which was available to the horses ad libitum. Each horse participated in a daily leisure workload at the riding school of the Didactic Stable. Daily leisure work included one hour of riding per day, five days a week. Additionally, all horses had access to a sandy paddock for six hours per day, seven days a week. Throughout the study period, horses continued their daily workload and free paddock activities.

### 2.2. Water Treadmill Exercise

Horses were exercised on a WT (Technohorse Sp. z o.o., Skarżysko-Kamienna, Poland) equipped with an AR system. The AR system used four water jets to create a continuous water flow at a speed of 3 m/s opposite to the horse’s movement (counterflow).

Prior to the data collection, each horse was equipped with a Polar Equine HR sensor (Polar Electro Oy, Kempele, Finland). The sensor was secured with an elastic belt around the thorax and placed on the left side of the horses’ chest. HR data from these sessions were reported in our recent related study [14].

Prior to the data collection, horses underwent a habituation protocol adapted from Greco-Otto et al. [13]. The habituation protocol included three initial WT exercise sessions (20 min duration per session): the first session on the treadmill with a dry belt, the second session in fetlock-depth water, and the third session in carpal-depth water. All habituation sessions were conducted were conducted at a walking speed of 1.25 m/s. During the last 5 min of the second and third sessions, the AR mode was activated. Horses were considered habituated when exhibited regular movement and no longer displayed any behavioral and HR-related signs of stress. Considering stress behaviors, snorting, whinnying, defecating, shaking, chewing, tail up or covered, and flared nostrils were assessed, while in HR variability, a significant increase in HR when starting to walk on the treadmill was considered. The ethogram of behavioral assessment was adapted from Maśko et al. [44], while the habituation protocol was adapted from Greco-Otto et al. [13]. After completing three habituation sessions, all horses were considered habituated.

The data collection was designed to include five WT exercise sessions (20 min duration per session, with an additional 5–10 min allocated for water filling and emptying, depending on water depth): the first session on the treadmill with a dry belt, the second and third in fetlock-depth water, and the fourth and fifth in carpal-depth water. During the third and fifth sessions, the AR mode was activated. Sessions were annotated as: dry treadmill (DT), fetlock-depth WT, fetlock-depth WT + AR, carpal-depth WT, and carpal-depth WT + AR sessions. The DT session served as the control. Each horse underwent one exercise session per day, and different session types were conducted on separate days, with the frequency of one session per week. All exercise sessions were conducted at a walking speed of 1.25 m/s. Water depth was adjusted individually for each horse. All sessions adhered to established guidelines for WT exercise in healthy horses [1]. The WT settings are summarized in Table 1.

### 2.3. Infrared Thermographic Imaging

#### 2.3.1. Thermographic Image Collection

Thermographic imaging was performed according to international guidelines for equine clinical practice [45]. Horses were imaged at the same time of day on consecutive days in June, with three horses imaged per day. Each horse was imaged twice—before and immediately after each WT session—resulting in a total of 150 images analyzed. All images were captured by the same researcher (U.S.).

The horses were brushed 1 h before imaging to remove dirt and mud and thus eliminate artifacts arising from surface emissivity variation. The horses were then led to an indoor hall equipped with a WT to acclimate to the imaging conditions. The indoor hall was a closed space, shielded from wind and direct sunlight. During imaging, the ambient temperature ranged from 20 °C to 25 °C (median: 23 °C), and relative humidity ranged from 50% to 64% (median: 55%). The water temperature ranged from 13 °C to 15 °C (median: 14 °C) for each WT session.

Thermographic images were taken from both the right and left sides of each horse using a non-contact thermographic camera (HIKMICRO SP60-L25, Hangzhou Microimage Software Co., Ltd., Hangzhou, China). The camera enables imaging with 640 × 480 (307,200 pixels) resolution and a spatial resolution of 0.66 mrad. The camera has a thermal sensitivity of <0.03 °C at temperatures of 30 °C (noise equivalent temperature difference (NETD) of <30 mK at 30 °C; F-number of the camera lens of 1.0), with a range of temperature detection between −40 °C and +150 °C. The emissivity (e) was set at 0.99. The camera has a manufacturer’s calibration certificate (Hangzhou Microimage Software Co., Ltd., Hangzhou, China). The camera was positioned approximately 2 m from the horse’s body to capture the entire lateral surface. The center of the field of view was aligned with the intersection of two reference lines—a horizontal line and a vertical line passing through the shoulder joint and the last rib, respectively.

#### 2.3.2. Thermographic Image Analysis

**Figure 1:**
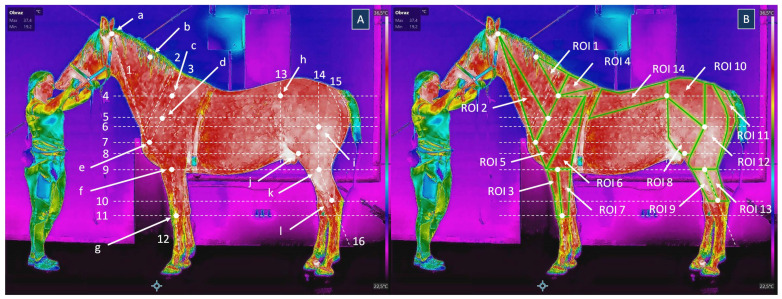
Thermographic images of the lateral surface of the same horse (**A**) with annotated landmarks and lines; (**B**) with annotated regions of interest (ROIs). Landmarks: a—wing of atlas, b—half the length of the neck, c—one third the length of the scapula, d—two thirds the length of the scapula, e—major tuberosity of humerus, f—lateral epicondyle of humerus, g—radiocarpal joint, h—tuber coxae, i—third trochanter of femur, j—patella, k—cut edge of deep crural fascia, l—tibiotarsal joint; Lines: 1—dorsal edge of the jugular groove, 2—cranial edge of the scapular muscles, 3—spine of the scapula, 4—horizontal level of one third of the scapula, 5—horizontal level of two thirds of the scapula, 6—horizontal level of the third trochanter of femur, 7—horizontal level of the major tuberosity of humerus, 8—horizontal level of the patella, 9—horizontal level of the lateral epicondyle of humerus and the cut edge of deep crural fascia, 10—horizontal level of the tibiotarsal joint, 11—horizontal level of the radiocarpal joint, 12—lateral midline of the forearm region, 13—vertical level of the tuber coxae, 14—vertical level of the third trochanter of femur, 15—the intermuscular groove (poverty line), 16—lateral midline of the leg region. The crosshair represents the lowest temperature on the image.

Thermographic images were processed using HIKMICRO Analyzer software, version 1.7.2 (Hangzhou Microimage Software Co., Ltd., Hangzhou, China). Each image was segmented into 14 regions of interest (ROIs), corresponding to body surface areas overlying 17 specific superficial muscles (Figure 1), as listed in Table 2. The consistent ROI placement across sessions and horses was ensured by using 12 landmarks (a–l) and 16 lines (1–16), shown in Figure 1, and determining the topographic location of selected muscles described in Table 2 [46]. The measurement areas consisted of the entire area of each ROI. All images were segmented by the same researcher (U.S.). ROIs were manually annotated on both the right and left sides of the horse’s body, resulting in a total of 4200 ROIs analyzed. For each ROI, Tmean and Tmax were measured. Tmean represented the mean temperature recorded over the entire area of each ROI, while Tmax represented the highest temperature recorded over the entire area of each ROI.

### 2.4. Statistical Analysis

Statistical analysis was performed using GraphPad Prism, version 6 (GraphPad Software Inc., San Diego, CA, USA). Data series were created by grouping surface temperature values (Tmean and Tmax) obtained from the right and left sides, and normality was assessed using the Shapiro–Wilk test. As not all data series followed a normal distribution, results are presented as medians and ranges (minimum and maximum values). Any ROI with wet hair coat after treadmill exercise was excluded from the analysis. Statistical significance was set at *p* < 0.05.

Tmean and Tmax data series were analyzed as paired data to assess the effect of exercise (comparing images before and after the treadmill session) for each ROI and session separately. If both data series were normally distributed, a paired *t*-test was used. If at least one data series was not normally distributed, the Wilcoxon matched-pairs signed-rank test was used.

Tmean and Tmax data series were analyzed as paired data to assess the effect of training type (comparing different sessions after exercise) for each ROI separately. When all data series were normally distributed, repeated measures ANOVA was used. If at least one data series did not follow a normal distribution, the Friedman test was used. When significant differences were found, post hoc tests were conducted—repeated measures ANOVA was followed by Holm–Šidák’s multiple comparisons test, while the Friedman test was followed by Dunn’s multiple comparisons test.

## 3. Results

### 3.1. Exercise Effect on Surface Temperature

**Figure 2:**
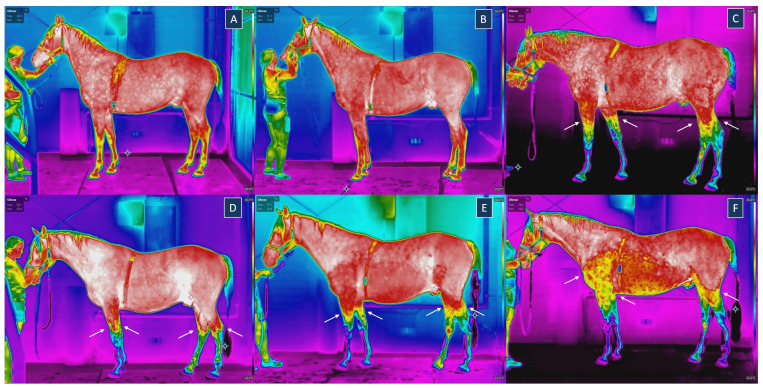
Thermographic images of the lateral surface of the same horse (**A**) before an exercise session; (**B**) after dry treadmill (DT) session; (**C**) after fetlock-depth water treadmill (WT) session; (**D**) after fetlock-depth WT session with artificial river (AR) mode; (**E**) after carpal-depth WT session; (**F**) after carpal-depth WT session with artificial river (AR) mode. Arrows indicate the upper level of wet hair coat after exercise. The crosshair represents the lowest temperature on the image.

Qualitative assessment of the thermographic images revealed the presence of wet hair coat above the level of the carpal joint following the fetlock-depth WT session and the fetlock-depth WT + AR session, in the vicinity of the elbow joint following the carpal-depth WT session, as well as above the level of the shoulder and knee joints after the carpal-depth WT + AR session (Figure 2). As a result, ROIs 3, 7, 9, and 13 were excluded from the post-exercise comparisons when WT at any depth was used. Additionally, ROIs 2, 5, 6, 8, 11, 12, and 13 were excluded from the post-exercise comparisons when carpal-depth WT + AR was used. In summary, 4 ROIs were excluded after the fetlock-depth WT, the fetlock-depth WT + AR, and carpal-depth WT sessions, while 10 ROIs were excluded after the carpal-depth WT + AR session. For all other ROI and session combinations, Tmean (Table 3) and Tmax (Table 4) were significantly higher (*p* < 0.0001) after exercise than before.

### 3.2. Training Type Effect on Surface Temperature

**Figure 3:**
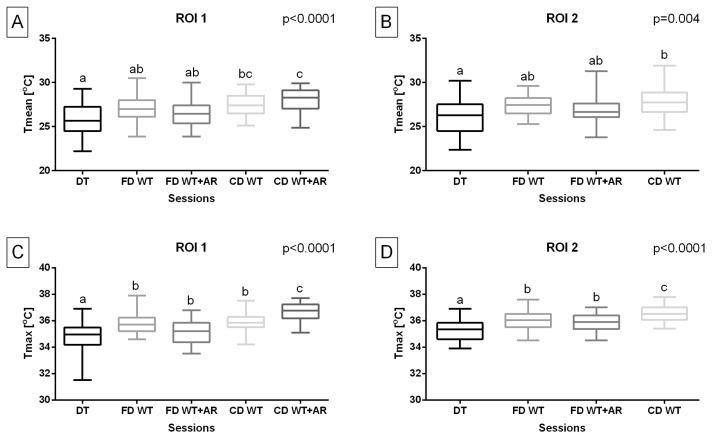
Training type effect on (**A**,**B**) the mean temperature (Tmean) and (**C**,**D**) the maximal temperature (Tmax) obtained from regions of interest (ROIs), representing body surface areas overlying specific superficial muscles responsible for forelimb protraction. The Tmean and Tmax were obtained after five sessions: dry treadmill (DT) session, water treadmill (WT) session in fetlock-depth water (FD WT), WT session in fetlock-depth water with artificial river (AR) mode (FD WT + AR), WT session in carpal-depth water (CD WT), and WT session in carpal-depth water with AR mode (CD WT + AR). Boxes represent median and lower and upper quartiles, while whiskers represent minimum and maximum values. Superscripts letters (a–c) indicate training-type-related differences. Statistical significance was set at *p* < 0.05.

Considering the temperature of the body surface overlying superficial muscles responsible for forelimb protraction, Tmean in ROI 1 was significantly higher (*p* < 0.0001) during both carpal-depth WT sessions compared to the DT session. Tmax in ROI 1 was significantly higher (*p* < 0.0001) during all WT sessions compared to the DT session as well as during the carpal-depth WT + AR session compared to other WT sessions. Tmean in ROI 2 was significantly higher (*p* = 0.004) during carpal-depth WT sessions compared to the DT session. Tmax in ROI 2 was significantly higher (*p* < 0.0001) during included WT sessions compared to the DT session and carpal-depth WT sessions compared to other included WT sessions. However, the carpal-depth WT + AR session was excluded from both temperature measures in ROI 2 (Figure 3).

**Figure 4:**
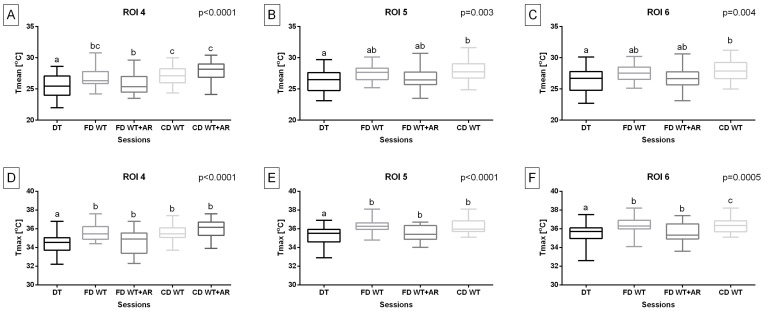
Training type effect on (**A**–**C**) the mean temperature (Tmean) and (**D**–**F**) the maximal temperature (Tmax) obtained from regions of interest (ROIs), representing body surface areas overlying specific superficial muscles responsible for forelimb retraction. The Tmean and Tmax were obtained after five sessions: dry treadmill (DT) session, water treadmill (WT) session in fetlock-depth water (FD WT), WT session in fetlock-depth water with artificial river (AR) mode (FD WT + AR), WT session in carpal-depth water (CD WT), and WT session in carpal-depth water with AR mode (CD WT + AR). Boxes represent median and lower and upper quartiles, while whiskers represent minimum and maximum values. Superscripts letters (a–c) indicate training-type-related differences. Statistical significance was set at *p* < 0.05.

Considering the temperature of the body surface overlying superficial muscles responsible for forelimb retraction, Tmean in ROI 4 was significantly higher (*p* < 0.0001) during all WT sessions compared to the DT session as well as during both carpal-depth WT sessions compared to the fetlock-depth WT + AR session. Tmax in ROI 4 was significantly higher (*p* < 0.0001) during all WT sessions compared to the DT session. Tmean in ROI 5 (*p* = 0.003) and ROI 6 (*p* = 0.004) was significantly higher during carpal-depth WT sessions compared to the DT session. Tmax in ROI 2 was significantly higher (*p* < 0.0001) during included WT sessions compared to the DT session and carpal-depth WT sessions compared to other included WT sessions. Tmax in ROI 5 (*p* < 0.0001) and ROI 6 (*p* = 0.0005) was significantly higher during all included WT sessions compared to the DT session. Moreover, Tmax in ROI 6 was significantly higher during carpal-depth WT sessions compared to both fetlock-depth WT sessions. However, the carpal-depth WT + AR session was excluded from both temperature measures in ROI 5 and ROI 6 (Figure 4).

Considering the temperature of the body surface overlying superficial muscles responsible for hindlimb protraction, Tmean in ROI 8 was significantly higher (*p* = 0.01) during carpal-depth WT sessions compared to the DT session. Tmax in ROI 8 was significantly higher (*p* = 0.002) during all WT sessions compared to the DT session. However, the carpal-depth WT + AR session was excluded from both temperature measures in ROI 8.

**Figure 5:**
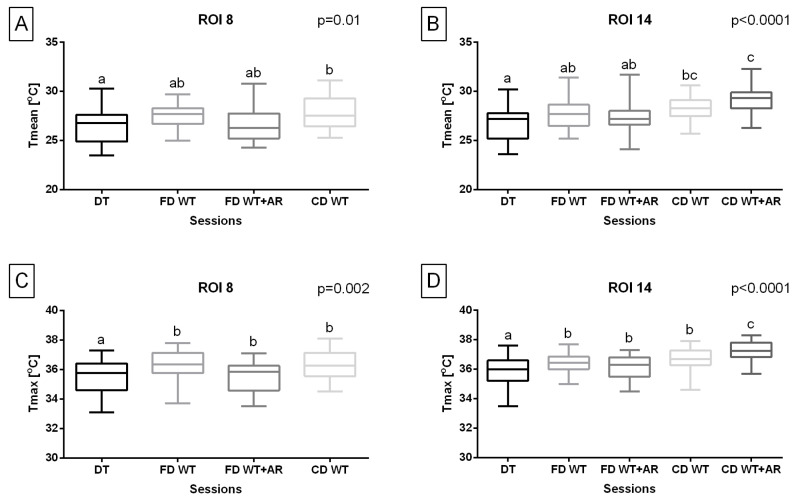
Training type effect on (**A**,**B**) the mean temperature (Tmean) and (**C**,**D**) the maximal temperature (Tmax) obtained from regions of interest (ROIs), representing body surface areas overlying specific superficial muscles responsible for (**A**,**C**) hindlimb protraction and (**B**,**D**) dorsoventral displacement of the trunk. The Tmean and Tmax were obtained after five sessions: dry treadmill (DT) session, water treadmill (WT) session in fetlock-depth water (FD WT), WT session in fetlock-depth water with artificial river (AR) mode (FD WT + AR), WT session in carpal-depth water (CD WT), and WT session in carpal-depth water with AR mode (CD WT + AR). Boxes represent median and lower and upper quartiles, while whiskers represent minimum and maximum values. Superscripts letters (a–c) indicate training-type-related differences. Statistical significance was set at *p* < 0.05.

Moreover, considering the temperature of the body surface overlying superficial muscles responsible for dorsoventral displacement of the trunk, Tmean in ROI 14 was significantly higher (*p* < 0.0001) during both carpal-depth WT sessions compared to the DT session as well as during the carpal-depth WT + AR session compared to both fetlock-depth WT sessions. Tmax in ROI 14 was significantly higher (*p* < 0.0001) during all WT sessions compared to the DT session as well as the carpal-depth WT + AR session compared to other WT sessions (Figure 5).

**Figure 6:**
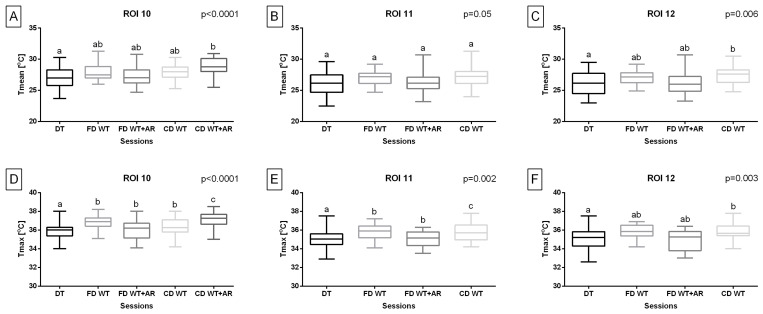
Training type effect on (**A**–**C**) the mean temperature (Tmean) and (**D**–**F**) the maximal temperature (Tmax) obtained from regions of interest (ROIs), representing body surface areas overlying specific superficial muscles responsible for hindlimb retraction. The Tmean and Tmax were obtained after five sessions: dry treadmill (DT) session, water treadmill (WT) session in fetlock-depth water (FD WT), WT session in fetlock-depth water with artificial river (AR) mode (FD WT + AR), WT session in carpal-depth water (CD WT), and WT session in carpal-depth water with AR mode (CD WT + AR). Boxes represent median and lower and upper quartiles, while whiskers represent minimum and maximum values. Superscripts letters (a–c) indicate training-type-related differences. Statistical significance was set at *p* < 0.05.

Considering the temperature of the body surface overlying superficial muscles responsible for hindlimb retraction, Tmean in ROI 10 was significantly higher (*p* < 0.0001) during the carpal-depth WT + AR session compared to the DT session. Tmax in ROI 10 was significantly higher (*p* < 0.0001) during all WT sessions compared to the DT session as well as during the carpal-depth WT + AR session compared to other WT sessions. Tmean in ROI 11 did not differ (*p* = 0.05) between treadmill sessions. Tmax in ROI 11 was significantly higher (*p* = 0.002) during all included WT sessions compared to the DT session as well as during carpal-depth WT sessions compared to both fetlock-depth WT sessions. Both Tmean (*p* = 0.006) and Tmax (*p* = 0.003) in ROI 12 were significantly higher during carpal-depth WT sessions compared to the DT session. However, the carpal-depth WT + AR session was excluded from both temperature measures in ROI 11 and ROI 12 (Figure 6).

## 4. Discussion

To date, only one study has been published on monitoring changes in body surface temperature associated with exercise on a conventional WT in horses [32]. The authors measured Tmax of the body surface overlying the *m. semitendinosus* before, during, and after 18 min of walking at a speed of 1.58 m/s on a DT, fetlock-depth WT, and carpal-depth WT. They demonstrated that, regardless of training type, Tmax of the body surface in the studied ROI increased at the onset of exercise and reached its highest value at the end of the session [32]. Our findings for the body surface overlying *m. semitendinosus* aligns with the results of the previous study. However, Yarnell et al. imaged the horses using IRT from a posterior–anterior view [32], whereas in the present study, IRT imaging was conducted from a lateral view. Consequently, while the ROIs included the body surface over the same muscle, they are not directly comparable. Additionally, in our study, horses walked for 20 min at a speed of 1.25 m/s, indicating a slightly different workload. One also may observe that Yarnell et al. [32] focused on only one superficial muscle, whereas our study examined 17 superficial muscles organized into 14 ROIs. Our findings for the body surface overlying all muscles studied, namely that the surface temperature increases with WT exercise, are consistent with the results reported by Yarnell et al. [32]. However, the main difference between the two studies lies in the use of the AR mode. Although both studies investigated a DT, fetlock-depth WT, and carpal-depth WT, our study is the first to demonstrate how the temperature of horses’ body surface changes when working on a WT with an AR.

However, regardless of whether IRT is considered a non-invasive tool for daily exercise monitoring on a conventional WT or a WT with an AR, this modality suffers from specific methodological limitations. IRT requires imaging of a clean and dry horse [34,45], as any dirt or moisture can affect the accuracy of surface temperature measurements. In the previous study [32], the authors stated that, during both the fetlock-depth WT and carpal-depth WT sessions, water only reached the level of the carpal joint. As a result, the body surface overlying the *m. semitendinosus* remained dry and was not affected by water, ensuring reliable surface temperature measuring. In this study, during the fetlock-depth WT and fetlock-depth WT + AR sessions, the water similarly reached only up to the carpal joint. However, during the carpal-depth WT session, water reached the level of the elbow joint, wetting areas of the body surface higher than in the previous study [32]. Therefore, even at relatively low water depths (up to the carpal joint) reliable temperature measurement of the body surface overlying the *m. extensor digitorum communis* [46] (a forelimb protractor [15]), the *m. extensor carpi ulnaris* [46] (a forelimb retractor [15]), the *m. extensor digitorum longus* [46] (a hindlimb protractor [15]), as well as the *m. flexor digitorum lateralis* [46] (a hindlimb retractor [15]) appears to be impossible. These muscles are involved in flexion and extension of the carpal and tarsal joints, respectively. Notably, flexion of the distal limb joints (metacarpophalangeal, carpal, and tarsal joints) has been shown to increase after WT exercise at low water depths [10,19,20,21]. Therefore, this kinematic effect cannot be indirectly monitored on a WT using IRT, which is a practical finding of this study. Furthermore, during carpal-depth WT + AR sessions, water reached the level of the shoulder and stifle joints. As a result, surface temperature data could not be reliably collected from the body surface overlying additional muscles, including a forelimb protractor (the *m. brachiocephalicus*), three forelimb retractors (the *m. deltoideus, m. infraspinatus,* and *m. triceps brachii*), two hindlimb protractors (the *m. quadriceps femoris* and *m. tensor fasciae latae*), and two hindlimb retractors (the *m. semitendinosus* and *m. biceps femoris*) [15,46]. This observation raises concerns about the validity of using IRT for indirect monitoring of muscle work and local blood flow during protraction and retraction of both the forelimbs and hindlimbs in carpal-depth WT + AR, and likely at higher water depths as well. Due to this methodological limitation, one may conclude that the utility of IRT for daily monitoring of individual training and rehabilitation programs on a WT may be limited when it comes to limb evaluation.

Given that extension and flexion of the distal limb joints—and thus protraction and retraction of the limbs—are of primary interest in studies on WT exercise [10,19,20,21], the question arises whether it is possible to minimize the wet coat effect and thereby expand the potential usefulness of IRT. Clipping the horse’s coat is a common practice in sport horses, as it shortens the hair length, thereby reducing sweat accumulation and decreasing drying time [47,48]. If horses are clipped before starting WT exercise, the drying time after each session is expected to be shorter. However, IRT imaging should be conducted on a dry horse [34,45], necessitating a delay between the end of WT exercise and the imaging procedure. On the other hand, IRT should be performed immediately after exercise [34,45], as the emission of thermal energy from the body surface causes post-exercise cooling and a reduction in surface temperature [38], which can significantly affect measurement results. Therefore, while comparing paired data from clipped horses before and after exercise is relatively straightforward (bearing in mind that changes in hair coat length affect IRT results [34,49]), the impact of drying time on temperature measurements requires further consideration and investigation. Accelerating the drying of the horse’s body surface may be possible through mechanical (manual) water removal. However, such mechanical friction may increase surface temperature and thus affect IRT measurements [50,51]. It is therefore recommended to brush the horse at least 1 h before IRT imaging [45]. Consequently, mechanical water removal immediately prior to IRT imaging is also contraindicated.

Yarnell et al. [32] reported a lower Tmax of the body surface overlying the *m. semitendinosus* after each WT session compared to the DT session. In contrast, our study found no differences in Tmax in this ROI and even showed a higher Tmax after each WT session compared to the DT session. Furthermore, in nearly all other ROIs—except the body surface overlying the *m. biceps femoris*—Tmax was also higher following WT sessions than DT sessions. Given that the increased extension and protraction of both forelimbs and hindlimbs against the resistance of water lead to greater muscle workload (depending on water depth [6,10,13,16,20,21,24,25,26] and belt speed [6,13,16,25,26]), an increase in body surface temperature with increasing workload was expected, as previously reported in dry treadmill [36,52,53] and overground studies [37,39]. The greater workload on the WT compared to the DT is supported by HR data from these horses, as reported in our recent related study, which showed that HR at the end of each WT session was higher than at the end of the DT session [14].

Yarnell et al. [32] speculated that the lower surface temperatures recorded during WT sessions, compared to DT sessions, could be attributed to the cooling effect of the water in the treadmill, which may reduce surface temperature and thus affect Tmax measurements. To date, only one study has investigated the effect of water temperature during WT exercise on blood circulation [27], but it did not assess body surface temperature. In this study, Nankervis et al. [27] speculated that a reduction in HR during the first 10 min of WT exercise in water at 13 °C, 16 °C, and 19 °C may result from enhanced heat dissipation. Notably, HR was significantly lower in 13 °C water than at the higher temperatures. The authors proposed that colder water might stimulate cutaneous cold receptors, influencing peripheral vascular resistance [27], leading to reduced peripheral blood flow and increased central blood flow [54,55]. They further suggested that WT exercise in water at temperatures of 19 °C or higher may promote heat storage [27], which may have appeared in some earlier WT studies where horses exercised in water at temperatures ranging from 20 °C to 27 °C [29,30,56,57,58]. In one study, the water temperature varied between 15 °C and 20 °C [20], while in others it was maintained between 13 °C and 15 °C [6,12,13,17,24] or even lower [8]. Similarly, in our study, water temperature was consistently maintained between 13 °C and 15 °C during each WT session [29]. In contrast, Yarnell et al. [32], like many other studies [3,4,5,7,9,10,11,14,16,21,23,25,26,28], did not report water temperature. Although the influence of water temperature on body surface temperature was not directly assessed in our and Yarnell et al.’s [32] studies, it should be considered a potential factor affecting IRT measurements. Moreover, when employing IRT, not only the water temperature but also the ambient temperature should be taken into account, given the high sensitivity of IRT to environmental conditions [34,45,49,59]. This aspect warrants further investigation, especially since most previous studies on equine WT exercise, aside from Yarnell et al. [32] and Nankervis et al. [27], do not report ambient temperature.

Yarnell et al. [32] reported no differences in Tmax of the body surface overlying the *m. semitendinosus* between fetlock-depth and carpal-depth WT sessions. In contrast, our study found that Tmax in the corresponding ROI was higher during carpal-depth WT sessions compared to both fetlock-depth sessions. This difference may result from the longer exercise duration and higher ambient temperature in our study compared to that of Yarnell et al. [32]. Similarly, a higher Tmax during carpal-depth WT sessions, relative to both fetlock-depth sessions, was observed for the body surface overlying the *m. brachiocephalicus* and *m. triceps brachii*. Additionally, a higher Tmax was recorded during carpal-depth WT + AR sessions than during other WT sessions for the body surface overlying the *m. trapezius pars cervicalis*, *m. gluteus superficialis*, *m. latissimus dorsi*, and *m. longissimus*. This increase in Tmax likely reflects greater heat production as a by-product of muscle contraction [35], thereby indirectly indicating higher muscle metabolism and increased local blood flow in the underlying tissue [33,34] and consequently greater workload of the respective muscles [36,37,38,39,40]. The fact that, as reported in our recent related study, HR at the end of carpal-depth WT sessions did not differ from either fetlock-depth WT session [14] suggests that at this level of workload the mechanism of higher muscle activity is more likely. However, the higher HR observed at the end of carpal-depth WT + AR sessions, compared to both fetlock-depth WT sessions [14], does not exclude the contribution of increased local blood flow required to meet the metabolic demands of active muscle fibers [41]—especially since blood lactate concentration was also elevated at this workload level [14].

The observed changes in Tmax suggest an increased intensity of *m. brachiocephalicus* activity during the carpal-depth WT session and of *m. trapezius pars cervicalis* during the carpal-depth WT + AR session. These findings align with previously reported kinematic data showing increased forelimb protraction while walking in carpal-depth water [10,20]. Additionally, the Tmax patterns indicate heightened activity of the *m. triceps brachii* and *m. semitendinosus* during the carpal-depth WT session, as well as increased activation of the *m. gluteus superficialis* during the carpal-depth WT + AR session, despite earlier reports of decreased forelimb [10,20] and hindlimb [10,17] retraction in this condition. However, these muscles also contribute to flexion–extension of the distal limb joints, which have been shown to increase during carpal-depth WT exercise based on kinematic analyses [10,19,20,21]. Moreover, walking in carpal-depth water has been associated with increased vertical displacement of the poll, withers, sacrum, and pelvis [5,10,18,21,22,23], as well as enhanced flexion–extension of the thoracolumbar spine [10,18,22] and pelvis [10,23]. These biomechanical adaptations may be reflected in the elevated Tmax observed over the *m. latissimus dorsi* and *m. longissimus*, indirectly suggesting an increased workload of these muscles during the carpal-depth WT + AR session.

Discussed observations of the level of hair coat wetting lead to another interesting conclusion. It appears that, when walking in carpal-depth water, the horses’ hair coat remained less wet when the AR mode was turned off compared to when it was turned on. Given that walking in carpal-depth water has been shown to increase forelimb and hindlimb protraction while decreasing retraction [10,17,20], it can be cautiously assumed that using the AR mode at this depth may further enhance limb protraction. This increased limb extension and protraction against water resistance could suggest a greater muscular workload. However, this hypothesis contradicts the findings of our previous study conducted on the same group of horses, which showed that HR was higher during the carpal-depth WT + AR session than during the DT, fetlock-depth WT, and fetlock-depth WT + AR sessions; however, no significant HR difference was observed between the carpal-depth WT sessions when the AR mode was turned off and turned on [14]. Therefore, further research is clearly needed to investigate the effect of AR on limb kinematics in horses [2,4,5,10,16,17,18,19,20,21,22,23,24,25,26].

## 5. Conclusions

Regardless of training type, surface temperature increases during WT exercise. However, its measurement in lateral thermographic images of horses is limited by the presence of a wet hair coat, which becomes more prominent as water depth increases from fetlock to carpal level and with the activation of the AR mode. Therefore, the utility of IRT for daily monitoring of individual training and rehabilitation programs on a WT may be limited when it comes to the indirect evaluation of limb muscle workload. Nonetheless, an increase in Tmax of the body surface overlying specific muscles—such as the *m. brachiocephalicus*, *m. trapezius pars cervicalis*, *m. triceps brachii*, and *m. semitendinosus*—may be suggested as a potential indirect indicator of increased activity related to forelimb protraction and flexion–extension of the limb joints during walking in carpal-depth water. Similarly, an increase in Tmax over the *m. latissimus dorsi* and *m. longissimus* may serve as a potential indirect indicator of increased vertical displacement of the trunk during carpal-depth WT exercise with active AR mode.

## Figures and Tables

**Table 1:** A summary of a treadmill settings used during exercise sessions: dry treadmill (DT) session, water treadmill (WT) session in fetlock-depth water, WT session in fetlock-depth water with artificial river (AR) mode, WT session in carpal-depth water, and WT session in carpal-depth water with AR mode.

WT Sessions	Duration	Belt Speed	Gait	Water Depth	AR Mode
DT	20 min	1.25 m/s	walk	dry belt	off
fetlock-depth WT	20 min	1.25 m/s	walk	fetlock level	off
fetlock-depth WT + AR	20 min	1.25 m/s	walk	fetlock level	on
carpal-depth WT	20 min	1.25 m/s	walk	carpus level	off
carpal-depth WT + AR	20 min	1.25 m/s	walk	carpus level	on

**Table 2:** A summary of regions of interest (ROIs), representing body surface areas overlying specific superficial muscles or muscle groups (*), along with a description of their topographical location following Ashdown and Done [46] as well as function following Nankervis et al. [10] and Krysiak et al. [15].

ROIs	Superficial Muscles *	Topographical Location	Function
ROI 1	*m. trapezius pars cervicalis*	from: half the length of the neck; to: one third the length of the scapula; at the level of spine of the scapula	forelimb protraction; scapula protraction
ROI 2	*m. brachiocephalicus*	from: wing of atlas; to: major tuberosity of humerus; dorsally to dorsal edge of the jugular groove	forelimb protraction; shoulder joint extension
ROI 3	*m. extensor digitorum communis*	from: lateral epicondyle of humerus; to: radiocarpal joint; cranially to the midline of the forelimb region	forelimb protraction; elbow joint flexion; carpal joint extension
ROI 4	*m. trapezius pars thoracica*	from: one third the length of the scapula; at the level of spine of the scapula; to: position of elastic belt	forelimb retraction; scapula retraction
ROI 5	*m. infraspinatus;* *m. deltoideus*	from: spine of the scapula; to: cranial border of *m. triceps brachii*; at the level of the lateral epicondyle of humerus	forelimb retraction; shoulder joint flexion
ROI 6	*m. triceps brachii*	from: the level of one third the length of the scapula; to: cranial border and caudal border of forelimb; at the level of lateral epicondyle of humerus	forelimb retraction; shoulder joint flexion; elbow joint extension
ROI 7	*m. extensor carpi ulnaris*	from: lateral epicondyle of humerus; to: radiocarpal joint; caudally to the midline of the forelimb region	forelimb retraction; carpal joint flexion
ROI 8	*m. quadriceps femoris*; *m. tensor fasciae latae*	from: tuber coxae and cranial border of hindlimb; to: the third trochanter of femur and the level of patella	hindlimb protraction; knee joint extension
ROI 9	*m. extensor digitorum longus*	from: cut edge of deep crural fascia; to: tibiotarsal joint; cranially to the midline of the leg region	hindlimb protraction; tarsal joint flexion
ROI 10	*m. gluteus superficialis*	from: level of the tuber coxae; to: level of the third trochanter of femur and line between tuber coxae and third trochanter	hindlimb retraction; hip joint extension
ROI 11	*m. semitendinosus*	from: intermuscular groove (poverty line); to: caudal border of hindlimb and the level of cut edge of deep crural fascia	hindlimb retraction; hip joint extension; tarsal joint extension
ROI 12	*m. biceps femoris*	from: caudal border of *m. gluteus* and *m. quadriceps*; to: intermuscular groove (poverty line) and the level of cut edge of deep crural fascia	hindlimb retraction; knee joint flexion
ROI 13	*m. flexor digitorum lateralis*	from: cut edge of deep crural fascia; to: tibiotarsal joint; caudally to the midline of the leg region	hindlimb retraction; tarsal joint extension
ROI 14	*m. latissimus dorsi* *;* *m. longissimus*	from: position of elastic belt at the level of two thirds the length of the scapula; to: level of the tuber coxae	dorsoventral displacement of the trunk

**Table 3:** The mean temperature (Tmean) (median and range (minimum; maximum)) obtained from regions of interest (ROIs), representing body surface areas overlying specific superficial muscles or muscle groups. The Tmean was obtained before and after exercise during five sessions: dry treadmill (DT) session, water treadmill (WT) session in fetlock-depth water, WT session in fetlock-depth water with artificial river (AR) mode, WT session in carpal-depth water, and WT session in carpal-depth water with AR mode. If any ROI included wet hair coat after treadmill exercise (wet), it was excluded from the comparison. Superscripts letters (a, b) indicate exercise-related differences. Statistical significance was set at *p* < 0.05.

Sessions	DT		Fetlock-Depth WT		Fetlock-Depth WT + AR		Carpal-Depth WT		Carpal-Depth WT + AR	
ROIs	Before	After	Before	After	Before	After	Before	After	Before	After
ROI 1	22.9 ^a^ °C (19.5; 26.2)	25.7 ^b^ °C (22.2; 29.3)	23.8 ^a^ °C (20.3; 26.5)	27.0 ^b^ °C (23.9; 30.5)	22.9 ^a^ °C (19.3; 26.7)	26.5 ^b^ °C (23.9; 30.0)	23.6 ^a^ °C (20.8; 25.6)	27.4 ^b^ °C (25.1; 29.8)	24.2 ^a^ °C (20.9; 25.9)	28.3 ^b^ °C (24.9; 29.9)
ROI 2	23.5 ^a^ °C (20.1; 27.0)	26.3 ^b^ °C (22.4; 30.2)	24.1 ^a^ °C (21.0; 26.6)	27.5 ^b^ °C (25.3; 29.6)	23.0 ^a^ °C (20.0; 28.3)	26.7 ^b^ °C (23.8; 31.3)	24.1 ^a^ °C (21.7; 27.0)	27.8 ^b^ °C (24.6; 31.9)	24.4 ^a^ °C (21.4; 26.6)	wet
ROI 3	23.0 ^a^ °C (19.7; 26.8)	25.9 ^b^ °C (22.6; 29.8)	23.5 ^a^ °C (20.9; 26.0)	wet	22.4 ^a^ °C (19.3; 27.8)	wet	23.3 ^a^ °C (20.9; 26.8)	wet	23.8 ^a^ °C (20.7; 26.2)	wet
ROI 4	22.2 ^a^ °C (18.7; 25.9)	25.5 ^b^ °C (22.0; 28.6)	23.4 ^a^ °C (20.8; 26.2)	26.4 ^b^ °C (24.2; 30.8)	21.9 ^a^ °C (18.4; 26.4)	25.4 ^b^ °C (23.5; 29.6)	23.3 ^a^ °C (19.9; 25.2)	27.1 ^b^ °C (24.4; 30.0)	23.5 ^a^ °C (20.2; 25.5)	28.2 ^b^ °C (24.1; 30.4)
ROI 5	23.9 ^a^ °C (20.2; 27.1)	26.5 ^b^ °C (23.1; 29.7)	24.2 ^a^ °C (21.8; 26.9)	27.7 ^b^ °C (25.2; 30.1)	23.0 ^a^ °C (20.0; 28.3)	26.5 ^b^ °C (23.5; 30.7)	23.9 ^a^ °C (21.5; 27.1)	27.8 ^b^ °C (24.9; 31.6)	24.5 ^a^ °C (21.7; 26.3)	wet
ROI 6	23.9 ^a^ °C (20.2; 27.1)	26.7 ^b^ °C (22.7; 30.1)	24.3 ^a^ °C (21.6; 26.8)	27.6 ^b^ °C (25.1; 30.2)	23.1 ^a^ °C (19.9; 28.6)	26.7 ^b^ °C (23.1; 30.6)	24.1 ^a^ °C (21.2; 27.8)	27.9 ^b^ °C (25.0; 31.2)	24.6 ^a^ °C (21.8; 26.7)	wet
ROI 7	22.9 ^a^ °C (19.5; 26.8)	26.2 ^b^ °C (22.6; 30.0)	23.4 ^a^ °C (21.1; 26.0)	wet	22.3 ^a^ °C (19.7; 27.3)	wet	23.3 ^a^ °C (20.8; 26.8)	wet	23.5 ^a^ °C (19.8; 26.2)	wet
ROI 8	23.6 ^a^ °C (21.4; 26.5)	26.8 ^b^ °C (23.5; 30.3)	23.8 ^a^ °C (20.7; 26.2)	27.7 ^b^ °C (25.0; 29.7)	22.9 ^a^ °C (19.0; 27.7)	26.3 ^b^ °C (24.3; 30.8)	23.5 ^a^ °C (21.4; 27.2)	27.6 ^b^ °C (25.3; 31.1)	23.8 ^a^ °C (22.1; 27.3)	wet
ROI 9	23.3 ^a^ °C (19.6; 26.8)	26.9 ^b^ °C (23.1; 29.8)	23.8 ^a^ °C (21.4; 26.5)	wet	22.4 ^a^ °C (19.3; 28.3)	wet	23.4 ^a^ °C (21.0; 27.8)	wet	23.7 ^a^ °C (21.1; 26.4)	wet
ROI 10	24.0 ^a^ °C (19.5; 26.8)	27.0 ^b^ °C (23.7; 30.3)	24.1 ^a^ °C (21.9; 27.3)	27.5 ^b^ °C (26.0; 31.3)	23.2 ^a^ °C (20.9; 26.5)	27.1 ^b^ °C (24.7; 30.8)	24.4 ^a^ °C (21.1; 26.3)	28.0 ^b^ °C (25.3; 30.3)	24.6 ^a^ °C (21.4; 26.6)	28.8 ^b^ °C (25.5; 30.9)
ROI 11	23.0 ^a^ °C (18.9; 26.1)	26.2 ^b^ °C (22.5; 29.6)	23.1 ^a^ °C (19.8; 24.9)	27.2 ^b^ °C (24.7; 29.2)	22.2 ^a^ °C (19.0; 27.7)	26.2 ^b^ °C (23.2; 30.7)	23.5 ^a^ °C (20.9; 27.2)	27.3 ^b^ °C (24.0; 31.3)	23.7 ^a^ °C (20.2; 25.8)	wet
ROI 12	23.3 ^a^ °C (19.7; 26.4)	26.2 ^b^ °C (23.0; 29.5)	23.6 ^a^ °C (20.9; 26.0)	27.2 ^b^ °C (24.9; 29.2)	22.4 ^a^ °C (19.5; 28.1)	26.0 ^b^ °C (23.3; 30.7)	23.5 ^a^ °C (20.4; 26.5)	27.6 ^b^ °C (24.8; 30.5)	23.9 ^a^ °C (20.7; 26.3)	wet
ROI 13	22.9 ^a^ °C (19.3; 26.6)	26.3 ^b^ °C (22.5; 29.2)	23.3 ^a^ °C (20.2; 25.5)	wet	21.6 ^a^ °C (19.2; 27.7)	wet	22.8 ^a^ °C (20.5; 25.4)	wet	23.1 ^a^ °C (20.8; 25.8)	wet
ROI 14	24.5 ^a^ °C (20.1; 27.7)	27.2 ^b^ °C (23.6; 30.2)	24.2 ^a^ °C (21.7; 27.6)	27.7 ^b^ °C (25.2; 31.4)	23.3 ^a^ °C (20.0; 28.3)	27.2 ^b^ °C (24.1; 31.7)	24.5 ^a^ °C (21.6; 26.8)	28.3 ^b^ °C (25.7; 30.6)	24.7 ^a^ °C (21.6; 27.1)	29.4 ^b^ °C (26.3; 32.3)
*p* value	<0.0001									

**Table 4:** The maximal temperature (Tmax) (median and range (minimum; maximum)) obtained from regions of interest (ROIs), representing body surface areas overlying specific superficial muscles or muscle groups. The Tmax was obtained before and after exercise during five sessions: dry treadmill (DT) session, water treadmill (WT) session in fetlock-depth water, WT session in fetlock-depth water with artificial river (AR) mode, WT session in carpal-depth water, and WT session in carpal-depth water with AR mode. If any ROI included wet hair coat after treadmill exercise (wet), it was excluded from the comparison. Superscripts letters (a–b) indicate exercise-related differences. Statistical significance was set at *p* < 0.05.

Sessions	DT		Fetlock-Depth WT		Fetlock-Depth WT + AR		Carpal-Depth WT		Carpal-Depth WT + AR	
ROIs	Before	After	Before	After	Before	After	Before	After	Before	After
ROI 1	33.2 ^a^ °C (31.1; 35.5)	35.0 ^b^ °C (31.5; 36.9)	34.1 ^a^ °C (31.6; 35.7)	35.7 ^b^ °C (34.6; 37.9)	33.1 ^a^ °C (30.7; 35.6)	35.2 ^b^ °C (33.5; 36.8)	34.1 ^a^ °C (31.7; 36.0)	35.9 ^b^ °C (34.2; 37.5)	33.8 ^a^ °C (32.0; 35.2)	36.8 ^b^ °C (35.1; 37.7)
ROI 2	33.6 ^a^ °C (31.0; 35.9)	33.8 ^a^ °C (32.4; 35.8)	33.8 ^a^ °C (32.4; 35.8)	36.1 ^b^ °C (34.5; 37.6)	33.4 ^a^ °C (31.4; 35.5)	35.9 ^b^ °C (34.5; 37.0)	34.2 ^a^ °C (32.6; 35.0)	36.5 ^b^ °C (35.4; 37.8)	34.1 ^a^ °C (32.9; 35.2)	wet
ROI 3	33.5 ^a^ °C (31.0; 35.5)	35.0 ^b^ °C (33.0; 36.5)	33.8 ^a^ °C (32.4; 35.6)	wet	32.9 ^a^ °C (29.7; 35.2)	wet	33.7 ^a^ °C (32.0; 35.3)	wet	34.1 ^a^ °C (31.3; 35.1)	wet
ROI 4	32.8 ^a^ °C (31.2; 35.2)	34.6 ^b^ °C (32.2; 36.8)	33.6 ^a^ °C (31.9; 35.3)	35.5 ^b^ °C (34.4; 37.6)	32.7 ^a^ °C (29.7; 35.0)	34.9 ^b^ °C (32.3; 36.8)	33.5 ^a^ °C (31.4; 35.8)	35.5 ^b^ °C (33.7; 37.4)	33.9 ^a^ °C (31.5; 35.2)	36.2 ^b^ °C (33.9; 37.6)
ROI 5	34.0 ^a^ °C (32.4; 36.3)	35.5 ^b^ °C (32.9; 36.9)	34.3 ^a^ °C (32.9; 36.1)	36.3 ^b^ °C (34.8; 38.1)	33.3 ^a^ °C (31.4; 36.1)	35.4 ^b^ °C (34.0; 36.7)	34.4 ^a^ °C (32.3; 36.0)	36.0 ^b^ °C (35.1; 38.1)	34.8 ^a^ °C (33.1; 35.9)	wet
ROI 6	34.2 ^a^ °C (32.3; 36.1)	35.7 ^b^ °C (32.6; 37.5)	34.3 ^a^ °C (33.1; 36.2)	36.3 ^b^ °C (34.1; 38.2)	33.5 ^a^ °C (31.3; 35.8)	35.3 ^b^ °C (33.6; 37.4)	34.7 ^a^ °C (32.4; 36.2)	36.4 ^b^ °C (35.1; 38.2)	34.8 ^a^ °C (32.8; 36.6)	wet
ROI 7	33.1 ^a^ °C (31.1; 35.4)	35.0 ^b^ °C (32.9; 37.1)	33.8 ^a^ °C (32.3; 35.6)	wet	32.8 ^a^ °C (29.8; 35.2)	wet	33.5 ^a^ °C (32.0; 35.0)	wet	33.9 ^a^ °C (31.4; 35.6)	wet
ROI 8	33.9 ^a^ °C (32.3; 35.9)	35.8 ^b^ °C (33.1; 37.3)	34.3 ^a^ °C (32.1; 35.7)	36.4 ^b^ °C (33.7; 37.8)	33.4 ^a^ °C (30.4; 36.1)	35.9 ^b^ °C (33.5; 37.1)	34.4 ^a^ °C (32.2; 36.0)	36.3 ^b^ °C (34.5; 38.1)	34.6 ^a^ °C (32.9; 36.5)	wet
ROI 9	33.6 ^a^ °C (31.3; 36.0)	35.5 ^b^ °C (32.4; 37.6)	34.1 ^a^ °C (32.8; 35.7)	wet	32.6 ^a^ °C (30.0; 35.5)	wet	34.0 ^a^ °C (31.7; 35.2)	wet	34.0 ^a^ °C (32.0; 35.3)	wet
ROI 10	33.9 ^a^ °C (32.0; 36.1)	36.0 ^b^ °C (34.0; 38.0)	34.6 ^a^ °C (33.0; 36.0)	36.9 ^b^ °C (35.1; 38.2)	33.5 ^a^ °C (31.9; 35.8)	36.2 ^b^ °C (34.1; 38.0)	34.5 ^a^ °C (32.7; 35.8)	36.3 ^b^ °C (34.2; 38.0)	34.7 ^a^ °C (32.5; 36.7)	37.3 ^b^ °C (35.0; 38.5)
ROI 11	32.9 ^a^ °C (31.2; 35.5)	35.1 ^b^ °C (32.9; 37.5)	33.4 ^a^ °C (31.8; 35.6)	35.9 ^b^ °C (34.1; 37.2)	32.7 ^a^ °C (30.4; 35.2)	35.2 ^b^ °C (33.5; 36.3)	33.8 ^a^ °C (31.8; 35.2)	35.7 ^b^ °C (34.2; 37.8)	33.9 ^a^ °C (32.2; 35.3)	wet
ROI 12	33.3 ^a^ °C (32.0; 35.6)	35.2 ^b^ °C (32.6; 37.5)	33.8 ^a^ °C (32.4; 35.8)	35.9 ^b^ °C (34.2; 36.9)	33.0 ^a^ °C (30.9; 35.3)	35.3 ^b^ °C (33.0; 36.4)	34.0 ^a^ °C (32.1; 35.6)	35.7 ^b^ °C (34.0; 37.8)	34.2 ^a^ °C (32.0; 35.5)	wet
ROI 13	32.7 ^a^ °C (31.3; 35.8)	35.0 ^b^ °C (31.8; 37.2)	33.1 ^a^ °C (32.1; 35.3)	wet	31.9 ^a^ °C (30.1; 34.9)	wet	33.4 ^a^ °C (31.2; 34.9)	wet	33.6 ^a^ °C (31.7; 35.1)	wet
ROI 14	34.5 ^a^ °C (32.4; 36.0)	36.0 ^b^ °C (33.5; 37.6)	34.8 ^a^ °C (33.1; 35.9)	36.5 ^b^ °C (35.0; 37.7)	34.3 ^a^ °C (31.4; 35.8)	36.3 ^b^ °C (34.5; 37.3)	35.0 ^a^ °C (32.6; 36.4)	36.7 ^b^ °C (34.6; 37.9)	35.1 ^a^ °C (33.7; 36.4)	37.3 ^b^ °C (35.7; 38.3)
*p* value	<0.0001									

## Data Availability

The data presented in this study are available upon request from the corresponding author.
